# Adiabatic excitation for ^31^P MR spectroscopy in the human heart at 7 T: A feasibility study

**DOI:** 10.1002/mrm.26576

**Published:** 2016-12-21

**Authors:** Ladislav Valkovič, William T. Clarke, Lucian A.B. Purvis, Benoit Schaller, Matthew D. Robson, Christopher T. Rodgers

**Affiliations:** ^1^ Oxford Centre for Clinical Magnetic Resonance Research (OCMR) University of Oxford Oxford United Kingdom; ^2^ Department of Imaging Methods Institute of Measurement Science, Slovak Academy of Sciences Bratislava Slovakia

**Keywords:** high energy phosphate, ^31^P‐MRS, ^31^P, heart, ultra‐high field, 7T, 7 Tesla, adiabatic

## Abstract

**Purpose:**

Phosphorus magnetic resonance spectroscopy (^31^P‐MRS) provides a unique tool for assessing cardiac energy metabolism, often quantified using the phosphocreatine (PCr)/adenosine triphosphate (ATP) ratio. Surface coils are typically used for excitation for ^31^P‐MRS, but they create an inhomogeneous excitation field across the myocardium, producing undesirable, spatially varying partial saturation. Therefore, we implemented adiabatic excitation in a 3D chemical shift imaging (CSI) sequence for cardiac ^31^P‐MRS at 7 Tesla (T).

**Methods:**

We optimized an adiabatic half passage pulse with bandwidth sufficient to excite PCr and γ‐ATP together. In addition, the CSI sequence was modified to allow interleaved excitation of PCr and γ‐ATP, then 2,3‐DPG, to enable PCr/ATP determination with blood correction. Nine volunteers were scanned at 2 transmit voltages to confirm that measured PCr/ATP was independent of $B_{1}^{+}$ (i.e. over the adiabatic threshold). Six septal voxels were evaluated for each volunteer.

**Results:**

Phantom experiments showed that adiabatic excitation can be reached at the depth of the heart using our pulse. The mean evaluated cardiac PCr/ATP ratio from all 9 volunteers corrected for blood signal was 2.14 ± 0.16. Comparing the two acquisitions with different voltages resulted in a minimal mean difference of −0.005.

**Conclusion:**

Adiabatic excitation is possible in the human heart at 7 T, and gives consistent PCr/ATP ratios. Magn Reson Med 78:1667–1673, 2017. © 2016 The Authors Magnetic Resonance in Medicine published by Wiley Periodicals, Inc. on behalf of International Society for Magnetic Resonance in Medicine. This is an open access article under the terms of the Creative Commons Attribution License, which permits use, distribution and reproduction in any medium, provided the original work is properly cited.

## INTRODUCTION

Phosphorus MR spectroscopy (^31^P‐MRS) allows noninvasive assessment of concentrations and/or reaction kinetics of high‐energy metabolites such as adenosine triphosphate (ATP) and phosphocreatine (PCr) in vivo 1, 2, 3, 4, 5. ^31^P‐MRS is of particular interest in cardiovascular medicine 6, as the PCr/ATP ratio in the heart changes in most major heart diseases, e.g., myocardial infarction 7, failing hypertrophied myocardium 8, or dilated cardiomyopathy 9, 10, where it can even serve as a predictor of mortality 11. The cardiac PCr/ATP ratio is also impaired in systemic diseases such as type 2 diabetes 12, 13 and in obesity 14.

Still, cardiac ^31^P‐MRS is not yet recognized as a practical tool for clinical applications. This is primarily because of the inherently low signal‐to‐noise ratio (SNR) for ^31^P‐MRS on clinical MR systems operating at less than or equal to 3 Tesla (T). To overcome this restraint, ultra‐high fields (e.g., 7 T), leading to more than doubled SNR 4, 15, 16, 17 and/or increased temporal resolution 18, are being used ever more often for ^31^P‐MRS. Further improvement in SNR for cardiac ^31^P‐MRS, accompanied with enhanced heart coverage, was recently demonstrated using a dedicated receive array combined with a single‐loop transmit radiofrequency (RF) coil at 7 T 19, 20.

Although achieving excellent receive sensitivity, the transmit performance ( $B_{1}^{+}$) of surface coils varies strongly depending on the exact position of the coil relative to the volume of interest, especially at ultra‐high fields. Accurate knowledge of flip angle (FA) in a given voxel is essential for reliable correction of partial saturation, which is required to determine metabolite concentrations or ratios 21. Computing field maps using quasi‐static approximation becomes increasingly inaccurate at ultra‐high fields, and direct measurement of FA maps requires additional acquisition time 22, 23, 24. The use of $B_{1}^{+}$‐insensitive adiabatic excitation providing uniform 90 ° excitation FAs over the whole heart, as demonstrated at lower fields 25, 26, would therefore constitute a significant improvement for cardiac ^31^P‐MRS at 7 T and provide an important step toward absolute quantification. However, application of adiabatic excitation pulses at 7 T is challenging because of their high $B_{1}^{+}$ requirements, high specific absorption rates (SARs), and because of the wide bandwidth (BW) of ^31^P‐MR spectra.

However, because the key metabolites of interest for cardiac ^31^P‐MRS are PCr and ATP, we hypothesized that a reduced excitation BW of the adiabatic half‐passage (AHP) pulse could be traded to decrease the required $B_{1}^{+}$ and SAR, while still exciting over a frequency range from PCr to γ‐ATP. This work benefits from the improved $B_{1}^{+}$ (≥16 μT) at the “depth of the heart,” i.e. at a distance of the intraventricular septum from the center of the coil (8–12 cm, depending on individual anatomy), provided by a purpose‐built quadrature ^31^P transceiver coil 27. Therefore, the aim of this study was to test the feasibility of a narrow‐band (∼300 Hz) adiabatic excitation for cardiac ^31^P‐MR 3D chemical shift imaging (CSI) at 7 T. In addition, to enable blood correction of the calculated PCr/ATP ratio, an interleaved acquisition of the 2,3‐diphosphoglycerate (2,3‐DPG) resonance frequency was implemented.

## METHODS

### Hardware

All measurements were performed on a 7T whole‐body MR system (Siemens Healthcare, Erlangen, Germany) equipped with a dual‐tuned (^31^P/^1^H) transmit‐receive quadrature, surface RF coil, consisting of a purpose‐built quadrature ^31^P coil (two 15‐cm loops, with overlap decoupling) and a single ^1^H loop (10 cm in diameter). The peak $B_{1}^{+}$ of this coil across the heart was estimated to be between 16 and 25 μT 27.

### Pulse Simulations and Sequence Design

**Figure 1:**
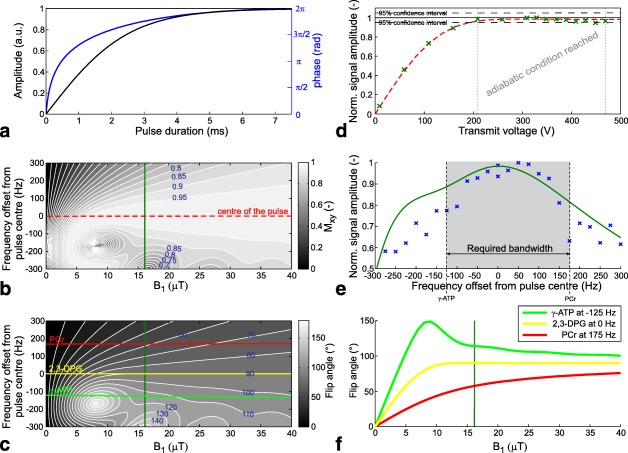
Simulations of the excitation pulse with embedded results from phantom experiments. (**a**) Amplitude and phase of the adjusted 7.5‐ms‐long tanh/tan AHP excitation pulse. (**b**) Bloch simulation of the amount of magnetization flipped to the xy plane after the AHP excitation pulse, the center of which (0 Hz) is shifted relative to PCr resonance frequency by −175 Hz in our in vivo experiments. The lower‐limit $B_{1}^{+}$ value expected for the used quadrature‐pair RF coil at the maximum voltage at the depth of the heart (i.e. 16 μT) is highlighted with a green line. (**c**) Simulated flip angles produced by the AHP pulse centered in vivo −175 Hz relative to PCr. The three lines represent the off‐resonance positions of the metabolites of interest: green, γ‐ATP ( −125 Hz); red, PCr (175 Hz) as compared with the yellow on‐resonance behavior expected for 2,3‐DPG during second acquisition. (**d**) Simulated dependence of the M_xy_ on the transmit voltage applied to the RF coil used, overlaid with the normalized signal amplitudes acquired in the localization phantom (green × ) at the depth of the heart. A 95% confidence interval delimits the plateau region where the amplitude is stable, indicating adiabatic 90 ° excitation. (**e**) Profile of the pulse simulated for a $B_{1}^{+}$ of 16 μT (green line) and as measured in the two‐compartment phantom at the depth of the heart (blue × ). The BW required to excite both PCr and γ‐ATP (i.e. ∼300 Hz at 7 T) is also depicted. Note that the BW is shifted from the center, ranging from −125 Hz (γ‐ATP) to 175 Hz (PCr), because of the asymmetry of the AHP pulse. (**f**) Flip angle as a function of $B_{1}^{+}$. Note the high stability of the flip angles for each metabolite (COV equal to 0.1, 2.7, and 5.6% for 0 Hz (2,3‐DPG), −125 Hz (γ‐ATP) and 175 Hz (PCr) excitation offset, respectively) for $B_{1}^{+}$ values expected in the heart (16–25 μT). This influences the saturation correction factors for γ‐ATP and PCr by less than 2.5 and 2.8%, respectively, given the ranges of flip angles, metabolite T_1_s, and the used TR.

A dedicated AHP excitation pulse (tanh/tan) 28 was adjusted using Bloch simulations through a manual tuning of its parameters (i.e. duration between 2.5 and 15 ms, ξ between 1 and 20, tan(κ) between 5 and 100 rad, and frequency sweep between 5 and 50 kHz). The main target was to obtain a low adiabatic threshold $B_{1}^{+}$ (<16 μT) and to minimize the SAR requirements, while maintaining a BW of at least 300 Hz at the depth of the heart. The resulting AHP pulse was 7.5 ms long, with its shape defined by ξ = 3 and tan(κ) = 85 rad and with a frequency sweep bandwidth of 25 kHz (Fig 1a). Bloch simulations of the excitation performance, e.g., magnetization in the xy plane (M_xy_) and FA of the selected pulse were performed for $B_{1}^{+}$ values from 0 to 40 μT using custom MATLAB (MathWorks, Natick, MA) routines. Figures 1b and 1c depict the M_xy_ and the excitation FA distribution of the selected AHP pulse, respectively. The dependence of M_xy_ on transmit voltage, visualized in Figure 1d, demonstrates the stability of M_xy_ value above the adiabatic threshold at 7 μT. The simulated off‐resonance excitation profile (i.e. BW) of the AHP pulse for the minimal $B_{1}^{+}$ expected at the “depth of heart” (16 μT) is depicted in Figure 1e.

Because of the narrowness of the designed AHP‐pulse BW at 7 T, only PCr and γ‐ATP resonance will be excited equally using this pulse. Although this is sufficient for straightforward PCr/ATP ratio evaluation, no information about the 2,3‐DPG signals, originating from the blood pool and necessary to correct for the blood‐ATP signal 29, would be available. Therefore, an ultra‐short echo time (UTE) 3D‐CSI 30 pulse sequence was modified to allow multiple excitations in an interleaved scheme. The excitation frequency offset was first targeted between the PCr and γ‐ATP resonances and then between the two peaks of 2,3‐DPG. As the BW of the AHP pulse is also asymmetric (Fig. 1e), the excitation frequency of the first acquisition was not centered exactly between the PCr and γ‐ATP signals, but instead was positioned closer to the γ‐ATP resonance frequency (i.e. at −175 Hz relative to the PCr). The excitation and acquisition at the second frequency (centered between the two peaks of 2,3‐DPG at 700 Hz) was performed in every other transient. As the off‐resonance phase of the pulse is nonlinear on one side, two‐step phase‐cycling was used to avoid disrupting the metabolites of interest during the other excitation.

### Phantom Experiments

The performance of the adjusted AHP pulse was tested using a two‐compartment phantom 4, which consists of an 18 L chamber filled with saline, providing coil loading similar to that of a lean volunteer, and a 2 × 2 × 2 cm^3^ cube filled with a solution of KH_2_PO_4_ doped with Gd‐DTPA (T_1_ = ∼1.7 s). The cube was fixed at a depth of 8 cm, simulating the position of myocardium in vivo, and the RF coil was placed on top of the phantom. Fully relaxed (repetition time (TR) = 10 s), nonlocalized, single‐average ^31^P‐MRS spectra were acquired on‐resonance using the UTE‐CSI sequence with the transmitter voltage increasing from 10 to 310 V in 50‐V steps, and above in 20‐V steps up to the maximum voltage of 470 V. The off‐resonance behavior of the AHP pulse was tested by acquiring ^31^P‐MRS spectra using the same sequence at the maximum voltage, by shifting the central excitation frequency in 25‐Hz steps from −300 to 300 Hz.

### In Vivo Experiments

Nine healthy volunteers (two females; age [mean ± standard deviation] 28.2 ± 5.3 years; body mass index (BMI) 22.8 ± 2.6 kg.m^−2^) were recruited and scanned in compliance with ethical and legal requirements. Subjects were measured in supine position, which was preferred over the prone position to maximize the volunteer comfort, even at the cost of increased breathing motion, with the RF coil positioned over their heart. The RF coil was tuned and matched before each examination to account for the potential differences in RF loading. Only small adjustments were needed between subjects, suggesting that coil loading and thus $B_{1}^{+}$ efficiency was similar for all subjects. CINE FLASH images were acquired using pulse oximeter gating (Siemens, München, Germany), and the UTE‐CSI matrix was then aligned to the short‐axis (SA) images. To test the adiabatic excitation in vivo, the cardiac 3D ^31^P UTE‐CSI scan using the interleaved excitation with the selected AHP pulse was repeated twice in each volunteer. First, at a transmit voltage giving 100% predicted SAR (i.e. ∼450 V) and then at 50 V less. This was also used to assess the scan‐to‐scan reproducibility. Because of SAR restrictions, relatively long TR (3000 ms) was mandatory between individual transients (the effective TR for each interleaved data set was therefore 6000 ms). Thus, to retain the acquisition time below 50 min, and to keep skeletal muscle contamination to a minimum, the spatial resolution in sagittal direction was sacrificed, yielding a matrix size of 8 × 16 × 8 (16 in the anterior–posterior direction) over a field of view of 240 × 240 × 200 mm^3^, using weighted acquisition with five averages in the center of k‐space. The acquisition BW of 6000 Hz was sampled by 1024 points. The resulting measurement time was 46 min and 42 s for each of the two UTE‐CSI acquisitions.

### Data Analysis

The amplitudes of the acquired phantom data were evaluated in MATLAB and compared to confirm the minimum transmit voltage giving 90 ° excitation on‐resonance at the depth of the heart. Similarly, the simulated excitation profile of the AHP pulse was validated using the amplitudes of the off‐resonance phantom measurements.

**Figure 2:**
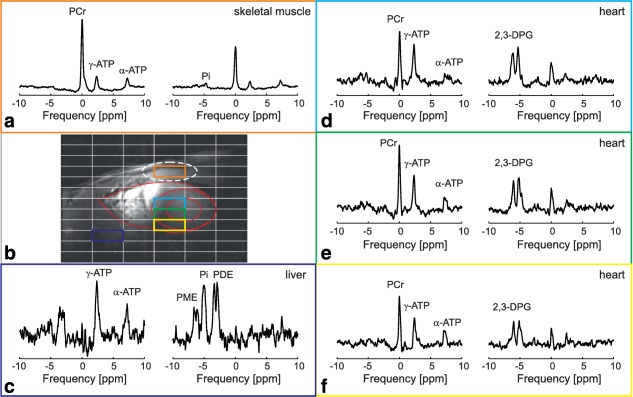
Typical acquired spectra from skeletal muscle (**a**), liver (**c**), and from three of the selected voxels from the heart septum (**d**–**f**). (**b**) Short‐axis 7T CINE FLASH localizer image showing the position of the selected voxels whose spectrum is plotted in the other panels. In panels showing ^31^P‐MR spectra, the spectra acquired with the excitation pulse centered at −175 Hz between PCr and γ‐ATP are depicted on the left, whereas spectra acquired with the excitation pulse centered at 700 Hz between the 2,3‐DPG resonance lines are depicted on the right. The heart is outlined in red. The simulated voxel size, affected by weighted acquisition, is depicted using a dashed white line.

For each subject, three septal voxels in the two mid‐SA slices (six in total) were selected for further analysis (Fig. 2b). Spectra from these voxels were phased to fix the first‐order phase, and fitted using a MATLAB implementation of the time‐domain fitting routine AMARES 31. The PCr and γ‐ATP signals, quantified from the first data set, were corrected for differences in T_1_ relaxation using the simulated off‐resonance FAs (Fig. 1f) and applying literature T_1_ values for cardiac ^31^P‐MRS at 7 T 4. The same correction factors were used for both UTE‐CSI experiments with different transmit voltages. The 2,3‐DPG signals were taken from the second data set and used to correct the calculated PCr/ATP ratio for blood contamination by subtracting 15% of the total 2,3‐DPG signal from the γ‐ATP signal 29.

To compare the two acquisitions with different transmit voltages and to demonstrate that the adiabatic onset has been reached in vivo at the lower voltage, Bland‐Altman analyses of agreement 32 were performed on the blood‐corrected PCr/ATP ratios; first for all 54 evaluated voxels and then for the mean PCr/ATP ratios from each of the nine volunteers. A two‐way random intraclass coefficient (ICC[2,2]) of absolute agreement 33 was evaluated for the mean blood‐corrected PCr/ATP ratios using SPSS (IBM SPSS, Chicago, IL) as a measure of reliability of the repeated measurement.

## RESULTS

The dependence of signal amplitudes of KH_2_PO_4_(aq), acquired in the phantom simulating the position of the myocardium, on the applied transmit voltage is depicted together with the theoretical values in Figure 1d. The amplitude increases steadily and then plateaus from 210 V upward. The off‐resonance performance of the AHP pulse as measured in the phantom is depicted in Figure 1e together with the BW predicted for comparable $B_{1}^{+}$.

Figure 2 depicts typical in vivo spectra acquired in skeletal muscle, liver, and in three of the selected voxels of the heart septum. Low contamination of the septal voxels by signal from the chest muscles is demonstrated. Because of the limited BW of the AHP pulse, the first interleaved spectra (left in each pair) contain only PCr, γ‐ATP signals, and a minor α‐ATP signal, whereas the second interleaved spectra (right in each pair) provide the 2,3‐DPG signals. Based on the Bloch simulations performed using the T_1_ of PCr at 7 T 4, the experimental TR and the expected $B_{1}^{+}$ in the myocardium, the M_xy_ of PCr after interleaved excitation is by less than 5% lower than the M_xy_ for a single excitation with a 6‐s TR. Details of this calculation are provided in the Appendix in the Supporting Information.

**Figure 3:**
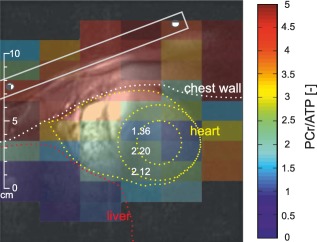
Typical PCr/ATP map, after blood correction, from the same volunteer depicted in Figure 2. The map shows reasonable consistency across the midsection of the LV (mean COV less than 27%). Chest muscles exhibit much higher PCr/ATP ratio (average 4.64 ± 1.10), and the liver tissue shows negligible PCr/ATP (average 0.51 ± 0.31).

**Figure 4:**
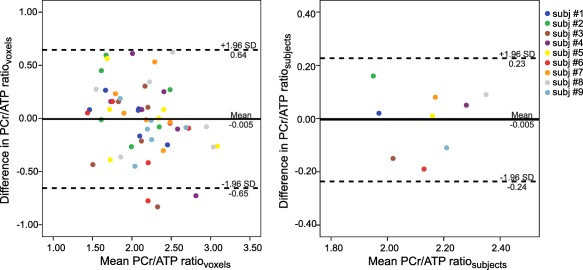
Bland‐Altman plots of the in vivo comparison between the scan acquired with maximum voltage applied and with 50 V less for the PCr/ATP ratio. Figure on the left depicts the comparison for all 54 selected voxels, and figure on the right shows the comparison for the volunteer means. The mean difference between the two scans is −0.005, showing only minimal bias. The repeatability coefficients are 0.65 and 0.23 for the voxel‐wise and volunteer‐wise analyses, respectively.

Figure 3 depicts a typical PCr/ATP map that is corrected for blood signal. A relatively consistent ratio across the whole left ventricle (LV) can be noticed. The mean intervoxel coefficient of variation (COV) through the LV beyond the selected voxels was 26.6 ± 3.9%. The mean PCr/ATP ratio calculated from the selected voxels was 2.13 ± 0.17 and 2.14 ± 0.17 for first and second UTE‐CSI acquisitions, respectively. The mean blood‐corrected PCr/ATP ratios determined for every volunteer from each UTE‐CSI acquisition are given in Table 1. Considerably higher values are present in the skeletal muscles of the chest wall (4.64 ± 1.10), whereas the liver region expresses minor PCr/ATP ratios (0.51 ± 0.31). The effective size of the ellipsoidal voxel 34 resulting from weighted acquisition, calculated at 64% of the main lobe 35 of the simulated point‐spread function, was 50.7 mL. Comparing the two UTE‐CSI acquisitions by the Bland‐Altman plots (Fig. 4), a mean bias of −0.005 could be identified for both cases (i.e. individual voxels and volunteer means). The ICC(2,2) of the absolute agreement between the results of the two UTE‐CSI acquisitions was 0.883.

**Table 1:** Mean Blood‐Corrected PCr/ATP Ratios of Each Volunteer Measured by Interleaved UTE‐CSI Sequence Using Two Different Transmit Voltages

	PCr/ATP		
Subject	MaxV	MaxV‐50V	Mean
**1**	1.96 ± 0.29	1.94 ± 0.43	1.95 ± 0.35
**2**	2.01 ± 0.37	1.85 ± 0.47	1.93 ± 0.38
**3**	1.93 ± 0.37	2.08 ± 0.38	2.00 ± 0.30
**4**	2.27 ± 0.28	2.23 ± 0.58	2.25 ± 0.39
**5**	2.14 ± 0.52	2.13 ± 0.64	2.13 ± 0.55
**6**	2.02 ± 0.45	2.20 ± 0.55	2.11 ± 0.47
**7**	2.19 ± 0.27	2.11 ± 0.35	2.15 ± 0.27
**8**	2.47 ± 0.59	2.38 ± 0.66	2.42 ± 0.61
**9**	2.22 ± 0.29	2.33 ± 0.31	2.28 ± 0.29
**Mean**	**2.13 ± 0.17**	**2.14 ± 0.17**	**2.14 ± 0.16**

Note: Values are given as the mean ( ± SD) of the six selected voxels of the heart for each individual subject and as the mean ( ± SD) of the volunteers for the mean value. The intrasubject SDs are influenced by partial overlapping of the neighboring voxels.

## DISCUSSION

In our study, we propose an interleaved UTE‐CSI sequence with adiabatic excitation at 7 T to measure PCr/ATP in the human heart. This approach allows adiabatic excitation, which makes the calculation of the blood‐corrected PCr/ATP throughout the sensitive volume of the coil more robust. The feasibility of the adiabatic excitation was investigated in phantom experiments and also in vivo in a group of healthy volunteers. High repeatability of the PCr/ATP measurement at 7 T was achieved with our technique, even though reduced transmit voltage was used in the second experiment.

In our localization phantom experiments, an increase in transmit voltage led, at first, to a steady increase in the acquired signal amplitude, which then plateaued. This indicated that an adiabatic threshold was reached and a $B_{1}^{+}$‐insensitive excitation was achieved with our AHP pulse at the depth of the heart at 210 V and above. Good agreement was found between the phantom results and the Bloch simulations. Similarly, the measured excitation profile of the AHP pulse agreed well with the simulated results. Although the BW of the AHP pulse is too narrow to excite the whole ^31^P‐MR spectrum at 7 T, for the $B_{1}^{+}$ values provided by our quadrature‐pair RF coil (i.e. over 16 μT at the depth of the heart), it does excite effectively over a bandwidth of 300 Hz, which is sufficient to excite both PCr and γ‐ATP. The excitation BW of our AHP pulse could be increased using frequency sweep cycling (FSC) as used by El‐Sharkawy et al. at 3 T 26. However, FSC requires paired acquisition for each phase‐encoding step of the CSI matrix, and therefore would significantly prolong the measurement time unless combined with a sparse k‐space sampling and non‐Fourier reconstruction.

To acquire the 2,3‐DPG signal, which is necessary for correction on the blood ATP signal, two consecutive CSI scans, first for PCr and γ‐ATP, and second for 2,3‐DPG, could be performed. However, we chose to implement an interleaved excitation scheme instead. Exciting PCr and γ‐ATP signals during odd transients and 2,3‐DPG in the even transients effectively means that the acquisition of the second data set is shifted in time by only one TR. This allows for quantification of all metabolites of interest under the same physiological conditions. Additionally, it also means that the interleaved approach effectively doubles the TR experienced by each metabolite, decreasing the influence of any uncertainties in the T_1_ used for saturation correction. This is of particular importance in patient studies as the pathology may alter the T_1_ values, as shown in the liver 36. If a simple, not interleaved, excitation experiment was performed using the effective TR of 6 s, the use of a more SAR‐demanding (e.g., longer) AHP pulse would be possible; however, adiabatic excitation of all metabolites of interest simultaneously would require a bandwidth of approximately 1000 Hz, which is more than three times as much as required for the presented approach. This could not be achieved for the $B_{1}^{+}$ range of our coil (16–25 μT), expected in the heart, by doubling or tripling the length of the AHP pulse. In addition, the unwanted T_2_ relaxation occurring during the pulse duration is inherently more pronounced in very long excitation pulses, which often makes them impractical.

Acquisition of good‐quality cardiac ^31^P‐MR spectra without contamination was achieved using the proposed approach. This was true also in segments of the LV beyond the septal voxels selected previously for further analysis, as the blood‐corrected PCr/ATP ratio was found to be reasonably consistent across the LV (mean COV less than 27%). Although the $B_{1}^{+}$ of the used RF coil unfortunately did not allow acquisition of ^31^P‐MR spectra from the whole heart, it was possible to cover the whole intraventricular septum and between one‐half and two‐thirds of the LV, depending on the individual heart orientation. In contrast to the myocardium, the chest muscles exhibited a higher PCr/ATP ratio, and the liver tissue showed negligible PCr/ATP ratios. This is encouraging, because there is no PCr present in healthy human liver. Consistent blood‐corrected PCr/ATP ratios across the heart provide further evidence of homogeneous excitation flip angle throughout the sensitive volume of the RF coil used. Looking at the actual blood‐corrected PCr/ATP in the heart, all calculated ratios for each volunteer, from both acquisitions as well as the mean value of 2.14 ± 0.16, fall inside the range of literature values in healthy volunteers 4, 10, 37, 38, 39.

Directly comparing the results of the two UTE‐CSI acquisitions for all 54 evaluated voxels by a Bland‐Altman analysis of agreement, a mean bias between the measurements of −0.005 and a repeatability coefficient of 0.65 could be identified. Furthermore, by comparing the mean PCr/ATP ratios of each volunteer between acquisitions (i.e. nine repeated measurements), the repeatability coefficient improved to 0.23. This represents higher repeatability than the previously reported interscan repeatability at 3 T, in which the PCr/ATP, averaged over three septal voxels, was measured in 20 healthy volunteers 39. In the aforementioned study, Tyler et al. 39 reported a mean interscan bias of −0.07 with a repeatability coefficient of 1.1. However, a larger nominal voxel size was used here (11.3 mL versus 5.6 mL in 39), and both acquisitions were performed within the same session. Nevertheless, this result supports our phantom data and indicates that we have exceeded the adiabatic onset in the heart in vivo.

A limitation of our method is the overall measurement time, caused by the relatively long TR (3 s) that is necessary to comply with the legal SAR limits, and because of the need to interleave the 2,3‐DPG acquisitions. Still, 3D‐CSI acquisition was possible in less than 47 min. Furthermore, the measurement time could be potentially reduced through shortening of the TR, as our results suggest that a lower transmit voltage would suffice for adiabatic excitation. Alternatively, faster localization strategies (e.g., one‐dimensional (1D) image selected in vivo spectroscopy (1D‐ISIS)) could be combined with the adiabatic excitation and interleaved acquisition scheme presented here. However, the volume selected by 1D‐ISIS depends on the sensitivity of the coil in the other two spatial directions. Therefore, 1D‐ISIS is more suitable for small‐diameter surface receive coils, as these are automatically insensitive to potentially contaminating tissue separated from the heart in the other two spatial directions. Our transmit/receive quadrature coil design provides the necessary $B_{1}^{+}$ at the depth of the heart, but its sensitivity volume in receive mode is too large to permit a clear‐cut selection of signal from the heart by 1D localization. Hence, we used a 3D‐CSI spatial‐encoding scheme in this study. Another limitation is the small residual PCr signal noticeable in the second data sets with excitation targeted at 2,3‐DPG. However, as this comprises less than 5% difference in M_xy_ using the interleaving against single excitation, its influence on the PCr/ATP ratio has been neglected. No cardiac gating was used in this study to avoid the possibility of bias due to increased miss‐triggering at 7 T 40. However, in future studies, acoustic cardiac gating 40 could be applied to reduce the cardiac motion influence on data quality.

In conclusion, our proof‐of‐concept study demonstrates that adiabatic excitation can be achieved in human heart at 7 T. We have inserted an AHP pulse with a relatively narrow BW and introduced interleaved excitation scheme into a UTE‐CSI sequence, enabling acquisition of consistent blood‐corrected PCr/ATP ratios. The use of a novel quadrature RF coil increased the available peak $B_{1}^{+}$, thus supporting the feasibility of adiabatic excitation at 7 T. This work paves the way for the first human studies with absolute quantification of cardiac ^31^P metabolites at 7 T.

## Supporting information


**Fig. S1**. Simulations of the M_xy_ under (**a**) ideal and (**b**) real steady‐state conditions for a given experimental TR and metabolite T_1_, as defined previously. (**c**) Simulated dependences of the Mxy on the $B_{1}^{+}$ for the resonance offset of PCr (175 Hz). The ideal steady‐state M_xy_ dependence is represented by a black line; the real steady‐state condition is represented by a blue line (full = odd and dashed = even transient). (**d**) Ratio of PCr M_xy_ excited during even and odd transients (green) and the percentage difference between the ideal and real (odd) M_xy_ of PCr (red).
**Appendix**. Quantification of the Influence of Partial PCr Excitation Using Interleaved Approach
