# Supplementary material for: Adiabatic excitation for 31P MR spectroscopy in the human heart at 7 T: A feasibility study
Source: Magn Reson Med. 2016 Dec 21;78(5):1667–73. doi: 10.1002/mrm.26576 (PMC5645675; doi:10.1002/mrm.26576)
Supplement: Supplementary file 1 — Fig. S1. Simulations of the Mxy under (a) ideal and (b) real steady‐state conditions for a given experimental TR and metabolite T1, as defined previously. (c) Simulated dependences of the Mxy on the B1+ for the resonance offset of PCr (175 Hz). The ideal steady‐state Mxy dependence is represented by a black line; the real steady‐state condition is represented by a blue line (full = odd and dashed = even transient). (d) Ratio of PCr Mxy excited during even and odd transients (green) and the percentage difference between the ideal and real (odd) Mxy of PCr (red). Appendix. Quantification of the Influence of Partial PCr Excitation Using Interleaved Approach [file MRM-78-1667-s001.docx]

Adiabatic excitation for
^31^P MR spectroscopy in the human heart at 7T:
 a feasibility study

**Supporting Information**

# Appendix SI1. Quantification of the influence of partial PCr excitation using the interleaved approach

As the excitation bandwidth of the used AHP pulse is too narrow (~300 Hz) to excite all the metabolites of interest, an interleaved excitation scheme was implemented to excite PCr and γ-ATP signals in odd transients and the 2,3-DPG signal in even transients. This, in ideal case, effectively doubles the TR experienced by each metabolite to 6 s. However, during the even excitations, the PCr resonance frequency is also partially excited.

In order to quantify the effect of this partial excitation on the quantitation of PCr signal from the odd acquisitions, the steady-state M_xy_ magnetization of myocardial PCr after the AHP excitation centred between PCr and γ-ATP was simulated numerically. Ideal (i.e. used during saturation correction) and real steady-state conditions were compared and their difference was quantified.

**Ideal conditions:**

- Single excitation or no excitation during the second excitation
- TR = 6 s (time between two excitations centred at the same resonance position)
- T_1_ = 3.05 s
- B_1_^+^ = 16 – 25 μT

**Real conditions:**

- Interleaved sequence with partial excitation during the second excitation
- TR = 3 s (time between two excitations centred at different resonance positions)
- The T_1_ and B_1_^+^ range are the same

The simulated steady-state M_xy_ magnetization under ideal and real conditions is depicted in Figure SI1a and SI1b, respectively. Figure SI1c depicts the comparison of dependences of M_xy_ on B_1_^+^ at the offset of PCr (175 Hz) between ideal (black) and real (blue) conditions. The full blue line represents the odd excitations and the dashed blue line even excitations of the interleaved sequence. Figure SI1d depicts the ratio of PCr M_xy_ excited during odd and even transients (green) and also the %difference between the ideal and real M_xy_ of PCr, expressed as a ratio of the absolute difference to the ideal condition (red).

For the range of B_1_^+^ expected in the heart, the difference does not constitute more than 5% difference between ideal and real conditions. Thus, the partial excitation of PCr in the interleaved approach influences the quantitation of myocardial PCr signal by less than 5%, under our experimental conditions.


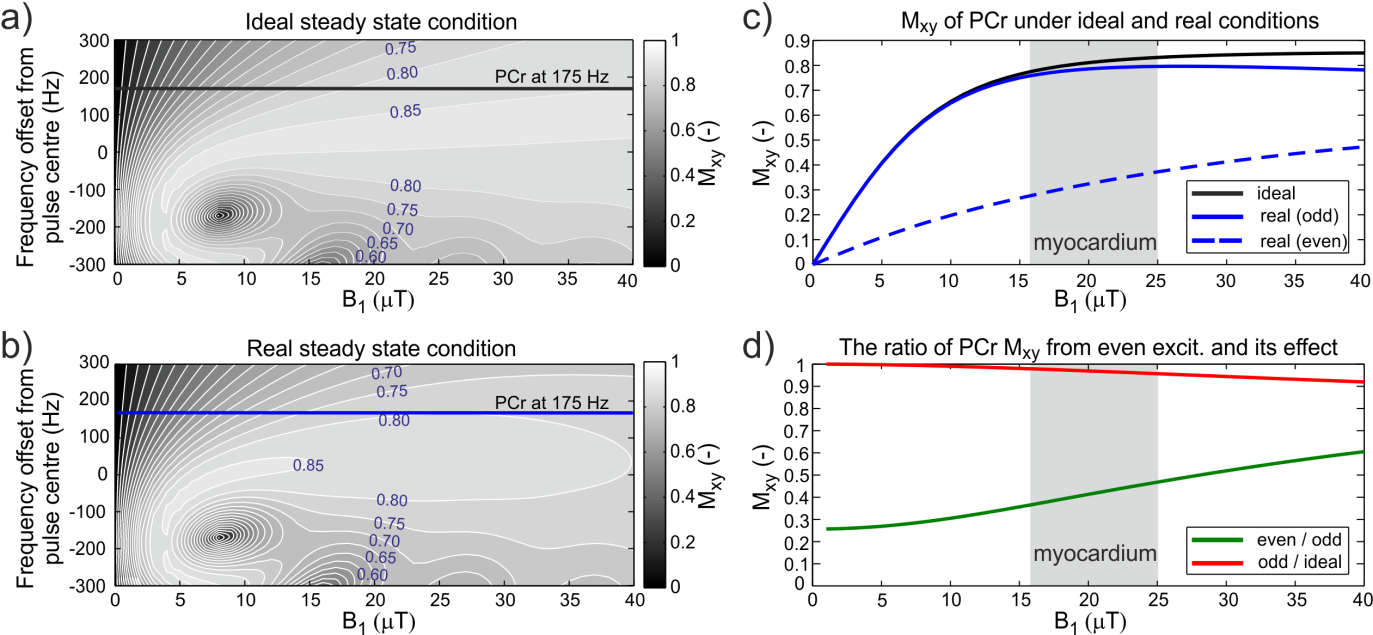


**Figure SI1** Simulations of the M_xy_ under (a) ideal and (b) real steady-state conditions for a given experimental TR and metabolite T_1_, as defined above. (c) Simulated dependences of the Mxy on the B_1_^+^ for the resonance offset of PCr (175 Hz). The ideal steady-state M_xy_ dependence is represented by a black line; the real steady state condition is denoted in blue line (full = odd and dashed = even transient). (d) Ratio of PCr M_xy_ excited during even and odd transients (green) and the %difference between the ideal and real (odd) M_xy_ of PCr (red).
